# Maternal ingestion of cocoa causes constriction of fetal ductus arteriosus in rats

**DOI:** 10.1038/s41598-021-89309-x

**Published:** 2021-05-11

**Authors:** Paulo Zielinsky, Felipe Villa Martignoni, Melissa Markoski, Kelly Pozzer Zucatti, Gabriela dos Santos Marinho, Gabriela Pozzobon, Pedro Rafael Magno, Victória de Bittencourt Antunes, Natassia Miranda Sulis, Alexandra Cardoso, Daniel Mattos, Alexandre Antônio Naujorks, Anize Delfino von Frankenberg, Izabele Vian

**Affiliations:** 1 Fetal Cardiology Unit, Institute of Cardiology/FUC, Avenida Princesa Isabel, 370, Porto Alegre, CEP 90620-000 Brazil; 2 Federal University of Santa Maria, Avenida Roraima, 1000, Santa Maria, CEP 97105-900 Brazil; 3 Departament of Pediatrics, Federal University of Rio Grande do Sul, Porto Alegre, Brazil; 4 Basic Health Science, Federal University of Health Sciences of Porto Alegre, Avenida Sarmento Leite, 245, Porto Alegre, CEP 90050-170 Brazil; 5 Fetal Cardiology Unit, Institute of Cardiology/University Foundation of Cardiology, Avenida Princesa Isabel, 395 – Santana, Porto Alegre, CEP 90620-000 Brazil

**Keywords:** Developmental biology, Cardiology, Medical research, Risk factors

## Abstract

Maternal consumption of polyphenol-rich foods has been associated with fetal ductus arteriosus constriction (DAC), but safety of chocolate exposure in fetal life has not been studied. This experimental study tested the hypothesis that maternal cocoa consumption in late pregnancy causes fetal DAC, with possible associated antioxidant effects. Pregnant Wistar rats, at the 21st gestational day, received by orogastric tube cocoa (720 mg/Kg) for 12 h, indomethacin (10 mg/Kg), for 8 h, or only water, before cesaren section. Immediately after withdrawal, every thorax was obtained and tissues were fixed and stained for histological analysis. The ratio of the narrowest part of the pulmonary artery to the fetal ductus inner diameter and increased ductal inner wall thickness characterized ductal constriction. Substances reactive to thiobarbituric acid were quantified. Statistical analysis used ANOVA and Tukey test. Cocoa (n = 33) and indomethacin (n = 7) reduced fetal internal ductus diameter when compared to control (water, n = 25) (p < 0.001) and cocoa alone increased ductus wall thickness (p < 0.001), but no change was noted in enzymes activity. This pharmacological study shows supporting evidences that there is a cause and effect relationship between maternal consumption of cocoa and fetal ductus arteriosus constriction. Habitual widespread use of chocolate during gestation could account for undetected ductus constriction and its potentially severe consequences, such as perinatal pulmonary hypertension, cardiac failure and even death. For this reason, dietary guidance in late pregnancy to avoid high chocolate intake, to prevent fetal ductal constriction, may represent the main translational aspect of this study.

## Introduction

Fetal ductal problems (premature closure, constriction, elongation, kinking and aneurysm formation) may have various cardiopulmonary consequences and—though symptoms and pathology are probably related to the type, fetal age, rapidity of progression and duration of intrauterine ductal dysfunction—can be potentially lethal^1^. Intrauterine constriction of the fetal ductus arteriosus (DAC) is a negative event that may lead to dilatation of the right ventricle, tricuspid insufficiency, pulmonary hypertension and potential development of fetal hydrops, heart failure and fetal or perinatal death^2–4^. Maternal ingestion of antinflammatory substances is the leading cause of DAC^5^ although up to 65% of cases were until recently considered of unknown origin^1^.

We have already reported that maternal ingestion of polyphenol-rich substances may be associated to fetal DAC, which is reversible after dietary restriction of these substances^2–4^. Additionally, events of idiopathic ductus arteriosus (DA) constriction and/or closure have been attributed to maternal consumption of polyphenol-rich foods in the late stages of pregnancy^4^.

The medical literature has widely described the effects of polyphenols on the adult vascular system and has scarcely studied its effect upon the fetus^5^. Nowadays, studies have shown that polyphenol-rich foods, as well as nonsteroidal anti-inflammatory drugs, may present risk to the fetus in late pregnancy^6^. Cocoa has the highest flavanol (epicatechin and catechin) content of all foods on a per-weight basis, being a significant contributor to total dietary intake of flavonoids^7^. Chocolate has been investigated in clinical settings to prevent preeclampsia, during the initial trimester of pregnancy, but its safety to the fetus has not been established^8–10^. Although metabolic derangements in offspring born to chocolate fed mothers have been reported in previous literature reviews^10^, the effects of this substance on the dynamics of DA are still unknown.

The purpose of this experimental study was to test the cause and effect relationship of maternal cocoa consumption in late pregnancy, resulting in fetal DAC. We also aimed to assess if DAC is accompanied by antioxidant effects in the mother and in the fetus.

## Methods

### Animals

Adult female Wistar rats (250–300 g) maintained under controlled conditions of light and environment (12-h light/dark cycle, 24 ± 1 °C, 55% relative humidity) with free access to food (Guabi, Santa Maria, Brazil) and water were used. *Ethics*: This study was conducted in accordance to the policies of the National Institutes of Health Guide for the Care and Use of Laboratory Animals (NIH Publications No. 80-23), revised in 1996, with the National Regulations for Animal Research (protocol number 0206) and performed in compliance with the ARRIVE guidelines 2.0 (*PLOS Biology*, https://doi.org/10.1371/journal.pbio.3000410). All procedures were conducted according to the Animal Experimentation Section of the Ethics Committee of Instituto de Cardiologia do Rio Grande do Sul. The study protocol was approved by the institutional Ethics Committee, according to the Brazilian Research Ethics Committee for the Use of Animals for Experimentation—*CEUA.* All efforts were made to reduce the number of animals used, as well as to minimize their suffering^9^.

Three nulliparas female rats were placed in cages with one male and left overnight. The day that vaginal plug and spermatozoa in the vaginal smear were detected was designated as day 0 of gestation and near-term was set at day 21. Each group was constituted of 4–8 litters and approximately 30–80 fetuses.

### Drugs

Indomethacin (Indocid, Aspen Pharma) was obtained from a local pharmacy. Baker´s cocoa powder (100%) was also purchased in a local market. Reagents were purchased from Sigma Pharma.

### Techniques

#### Drug administration protocol

The protocol chosen in this study for drug administration was based on the fact that, to induce pharmacological constriction of the ductus by prostaglandin synthesis inhibition, the drug must be administered acutely near term^11^.

The doses of cocoa of 7.2, 72 or 720 mg/Kg were established based on a clinical study which determined dark chocolate consumption among third trimester pregnant women from the general population, on the 25th, 50th and 75th percentiles, relating the ranges of consumption to their average weight^4^. In the present study, we established a percentual relation with the pregnant rats average weights, for the three percentiles, at the equivalent period of gestation.

The effect of cocoa and indomethacin on DA was investigated by administration through orogastric tube with a 1 ml suspension of tap water with cocoa (7.2, 72 or 720 mg/Kg) for 12 h, tap water with indomethacin (10 mg/Kg) for 8 h or tap water only (1 mL) for 8 h before the surgical procedure. All drugs were administered over a 1-min period using a 1 mL insulin syringe. The doses of indomethacin and time elapsed between drug injection and surgical procedure were selected based on previous studies^11^.

#### Cesarean section

All pregnant rats were anesthetized at the near-term 21st day of gestation with ketamine (80 mg/Kg)/xylazine (8 mg/Kg, i.p.), after having received cocoa, indomethacin or water, as described above, and placed at the operating table. Indomethacin, because its well known constrictive effect on the ductus arteriosus, was considered as the positive control and water the negative control for DAC. The thorax from each fetus was obtained immediately after caesarean section and before the first breath. Fetuses were delivered quickly and fixed within 5 s after delivery by the rapid whole-body freezing technique using acetone cooled to − 80 °C by dry ice. Body weight of fetuses was measured in the frozen state to assess fetal maturation. The thorax of the frozen fetus was trimmed and sectioned on the freezing microtome to obtain transverse sections of the main pulmonary artery (PA) and the ductus arteriosus (DA)^12–16^.

Tissues were fixed in 10% neutral formaldehyde for 48 h, dehydrated with increasing concentrations of ethanol and embedded in paraffin. Serial sections the torso of each embryo were taken, stained with hematoxylin–eosin, and the appropriate areas were photographed.

#### Measurement of fetal ductus arteriosus

**Figure 1:**
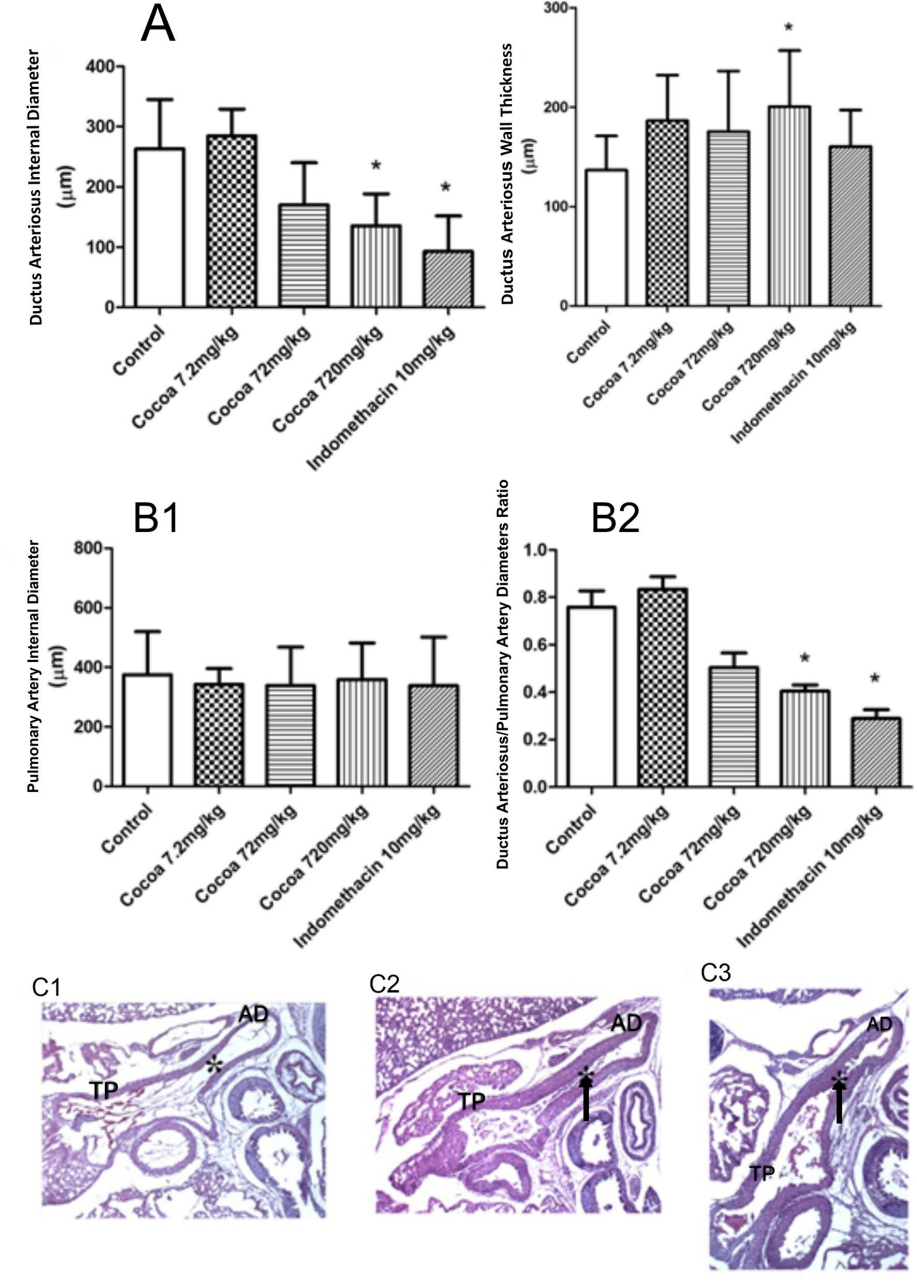
(**A**) Effects of cocoa on DA internal diameter and DA wall thickness. Cocoa (720 mg/Kg, p.o. n = 33) and indomethacin (10 mg/Kg p.o. n = 7) caused fetal DAC, but cocoa (720 mg/Kg, p.o.) alone increase ductus wall thickness*.* Lower doses of cocoa had no effect over the DA (7.2 mg/Kg n = 4 and 72 mg/Kg n = 4). *p < 0.001 compared with vehicle group (ANOVA). Data are mean ± standard deviation for *n* = 7–33 in each group. (**B**) Effects of cocoa on pulmonary artery internal diameter and ratio of the DA internal diameter and fetal pulmonary artery internal diameter. Vehicle (n = 25), cocoa (7.2 mg/Kg p.o. n = 4. 72 mg/Kg p.o. n = 4 and 720 mg/Kg, p.o. n = 33) and indomethacin (10 mg/Kg p.o. n = 7) showed no difference in pulmonary artery diameter (**B1**), but cocoa (720 mg/Kg, p.o. n = 33) and indomethacin (10 mg/Kg i.p. n = 7) decreased DA/PA ratio (**B2**). *p < 0.001 compared with vehicle group (analysis was made by one-way ANOVA, the differences between groups confirmed by Tukey´s Multiple Comparison test). Data are mean ± standard deviation for *n* = 7–33 in each group. (**C**) Representative fetal tissue histology of sagittal cross sections of the main pulmonary artery, ductus and descending aorta. These three cross sections of the heart were recorded at the same magnification (× 10 objective lens). The fetus ductus arteriosus was well patent in the control heart (**C1**). Twelve hours after the administration of cocoa 720 mg/Kg through gastric gavage (**C2**) or 8 h after p.o. administration of indomethacin 10 mg/Kg (**C3**) the fetus ductus arteriosus was significantly constricted. *TP* trunk of pulmonary artery;  *AD* arterial duct*.*

The inner diameters of each fetal DA and pulmonary artery diameters were photographed using an Olympus photomicroscope with a 10X objective lens and a digital camera. The images were digitalized and diameters measured by ImageJ software. The mean fetal DA and pulmonary artery diameters were determined in micrometers and the ratio of the narrowest part of the pulmonary artery (PA) to the fetal DA (DA/PA) was considered for the diagnosis of ductal constriction (Fig. 1). Ductal wall thickness was measured, but was not considered an essential parameter for ductal constriction, due to its expected differences along the ductal length.

### Data analysis

Data are expressed as mean ± S.E.M. The differences among groups were assessed using analysis of variance (one way ANOVA). If a significant difference among groups was demonstrated, Tukey´s Multiple Comparison test was applied. *P* value of less than 0.05 was considered significant.

### Liver homogenization

The liver tissue was homogenized (1:10 w/v) with Tris–HCl buffer 50 mM pH 7.5 and centrifuged at 800 g for 10 min at 4 °C. The supernatant was used to perform oxidative stress assays^17^.

### Protein determination

Protein was measured by the Coomassie blue method by using serum albumin as standard^18^. To measure protein, the maternal homogenized liver supernatant was further diluted to reach 1:40 titration and fetal homogenized liver was diluted to reach 1:20 titration.

### Thiobarbituric reactive substances

As an index of lipid peroxidation, the formation of thiobarbituric reactive substances (TBARS) during an acid heating reaction was used as already described; 200 µL of homogenized tissue supernatant (1:10 w/v) samples were mixed with 500 µL of acetic acid (2.5 M pH 3.4), 500 µL of TBA (0.8%), 200 µl of sodium dodecyl sulphate (SDS; 8.1%) and 100 µL of distilled water^19^. Subsequently, they were heated in boiling water bath for 120 min. TBARS were determined by the absorbance at 532 nm and were expressed as malondialdehyde equivalents.

### SOD and CAT activities

The homogenized tissue supernatant (1:10 w/v), was used to determine superoxide dismutase activity (SOD). To determine catalase enzymatic activity (CAT), the homogenized tissue supernatant was further diluted to reach a 1:60 titration. CAT enzymatic activity was measured by the rate of decrease in H_2_O_2_ at 240 nm^20^. SOD activity was assayed by measuring the inhibition of adrenaline auto-oxidation as absorbance at 480 nm^21^.

## Results

Maternal ingestion of cocoa (720 mg/Kg, p.o. n = 33) and indomethacin (10 mg/Kg p.o. n = 7) reduced fetal internal DA diameter when compared to control (water, p.o. n = 25), (mean: 263 ± 81 µm × 135 ± 53 µm and 263 ± 81 µm × 92 ± 5 µm, respectively, *P* < 0.001) (Fig. 1A). Lower doses of cocoa (7.2 mg/Kg n = 4 and 72 mg/Kg n = 4) had no effect on the DA (Fig. 1A). Similarly to indomethacin, cocoa decreased the ratio between ductal internal diameter and pulmonary artery internal diameter compared to control group (0.4 ± 0.1 and 0.2 ± 0.09 × 0.72 ± 0.3, respectively, *P* < 0.001) (Fig. 1B2). Sagittal cross sections of the fetal heart showed DAC after administration of both cocoa and indomethacin compared with the control group ((Fig. 1C1−C3). Cocoa alone increased DA wall thickness, when compared to indomethacin and control (200 ± 56 µm × 160 ± 36 µm × 136 ± µm, respectively, *P* < 0.001), (Fig. 1A). On the other hand, cocoa did not alter pulmonary artery diameter when all groups were compared (Fig. 1B1). We also tested whether the constrictive effect of cocoa was related to its antioxidant properties. On Table 1 we show that cocoa ingestion 12 h before labor did not alter SOD and CAT activities or TBARS concentrations on fetal and maternal livers.

**Table 1:** Effect of Cocoa (720 mg/Kg; p.o.) and indomethacin (10 mg/Kg p.o.), on fetal liver oxidative stress and maternal.

Treatment	Vehicle (n = 4)	Cocoa (720 mg/kg, n = 7)	Indomethacin (10 mg/kg, n = 6)	p
**Fetal liver oxidative stress**				
^†^CAT (nmol/mg protein)	9.86 ± 5.87	8.98 ± 5.55	10.14 ± 3.68	0.50
^‡^SOD (U SOD/mg of protein)	9.03 ± 5.88	7.67 ± 2.84	6.24 ± 2.09	0.53
^§^TBARS (ng/mg protein)	14.83 ± 3.55	15.68 ± 4.40	13.89 ± 6.55	0.87

Treatment	Vehicle (n = 5)	Cocoa (720 mg/Kg, n = 6)	Indomethacin (10 mg/Kg, n = 8)	p
**Maternal liver oxidative stress**				
^†^CAT (nmol/mg protein)	12.61 ± 9	15.18 ± 1.28	13.89 ± 1.33	0.25
^‡^SOD (U SOD/mg of protein)	26.07 ± 27.54	16.01 ± 9.99	9.06 ± 6.89	0.31
^§^TBARS (ng/mg protein)	3.65 ± 4.4	2.52 ± 1.43	2.77 ± 1.02	0.46

^†^Data are mean ± standard deviation range of ^†^catalase activity (CAT), ^‡^superoxide dismutase activity (SOD).

^§^Thiobarbituric reactive substances (TBARS) of cocoa compared with vehicle-indomethacin group (analysis was made using one-way ANOVA, p > 0.05).

## Discussion

In the present experimental study, we show that in the last third of pregnancy (period equivalent to human third trimester), gastric administration to the mother of cocoa, 720 mg, which corresponds to 0.7 g/kg of 100% cocoa chocolate, caused fetal DAC. Once established, this effect persisted up to the moment of pregnancy interruption and occurred without altering conventional oxidative stress markers in fetal or maternal tissues.

Cocoa has the highest flavanol (epicatechin and catechin) content of all foods on a per-weight basis, being a significant contributor to the total dietary intake of flavonoids^7^. Meta-analyses showed that cocoa consumption decreases multiple cardiovascular risk factors in adults, such as blood pressure, insulin resistance, lipid profiles, and flow-mediated vascular dilatation (FMD)^7,22–24^. Additionally, cocoa products significantly increase endothelium-derived nitric oxide (NO) synthesis and metabolism^25^. We have already demonstrated that chronic supplementation of polyphenols in the last third of pregnancy decreased two–threefold NO levels and plasma oxidative stress markers and had strong correlation to fetal DAC in sheep^26^.

In contrast to previous studies that showed that even a single dose of chocolate/cocoa may decrease oxidative stress markers in human peripheral blood, this outcome was not demonstrated in the rat liver^25^. It is important to stress that liver was the target tissue used in this study because of its easy accessibility and its role in polyphenol metabolism humans and animals^27^. In human fetuses, we have previously demonstrated reversal of ductal constriction after 2 weeks of nutritional guidance to reduce maternal consumption of polyphenol-rich foods in the third gestational trimester^3^ and that this reversal resulted in increase of prostaglandin E2 plasma level^28^. We have also shown that chronic administration of green tea caused oxidative stress markers decrease and fetal DAC in sheep^2^.

In this animal study we show the constrictive action of cocoa upon the fetal ductus arteriosus when ingested by the mother in the last third of gestation, mimicking the well established effect of indomethacin, a nonsteroidal anti-inflammatory drug with potent prostaglandin inhibitor action. It is noteworthy that the widespread consumption of chocolate by the general population, since it was habitually considered safe in pregnancy and even been used in clinical trials to prevent preeclampsia, may have potential harmful effects on the fetus, as herein shown and similar to other polyphenol-rich foods and beverages^8,29–32^.

In relation to the possible dose–effect relationship of polyphenol ingestion in pregnancy, we have already published a clinical study showing that polyphenol consumption by third trimester pregnant women above the 75th percentile caused alterations in fetal ductus arteriosus dynamics when compared to fetuses exposed to a polyphenol maternal ingestion below the 25th percentile^4^.

One feature that should be pointed out in this study is that significant increase in wall thickness has occurred only with 720 mg/kg of cocoa, but lower doses of cocoa and indomethacin have shown some increase comparing to water, albeit with no significance. It has been shown that the decrease in ductal inner diameter which characterizes ductal constriction may coexist with different degrees of diffuse or localized increase in wall thickness along the ductal lenght, what could account for the different degrees of wall thickness observed in the present study^33^.

In the present experiment, we have used a cocoa concentration of 100%, thus without any other substances other than polyphenols. The largest group of phenolic compounds found in raw cocoa beans are flavanols, represented primarily by flavanols, catechin and anthocyanins. Other phenolic compounds present in the beans include mainly quercetin and its glycosides. It has not been shown degradation of polyphenolic compounds in beans and nibs, when roasted under constant process parameters^34^. Another report has shown that cocoa present in powder and chocolate, as well as its total polyphenols, flavan-3-ol monomers and procyanidins, are stable over time^35^.

A potential limitation of this study could be the lack of assessment of inflammatory factors, such as prostaglandin E2, whose inhibition has a known cause and effect relationship with ductal constriction. Nevertheless, this fact has been previously demonstrated in clinical and experimental studies, which showed that prostaglandin E2 increases after reversal of ductal constriction related to maternal ingestion of polyphenol-rich substances^11,28^, as well as in vitro research studies demonstrating inhibitory activity of cocoa phenols on the prostaglandin E2 axis and production^36^ as well as the anti-inflammatory effect of dark chocolate due to the action of its polyphenols through modulation by NF-kB, which inhibits prostaglandin production^37^. In relation to oxidative stress and absence of antioxidant effects of cocoa before and after ductal constriction, it is in contrast to other studies which have shown decrease in TBARS after polyphenol supplementation in late pregnancy^26^, and vasoconstricting response in fetal ductal constriction, due to endothelin-1 mediated decrease in nitric oxide synthase activity^38^.

In addition to clinical and experimental reports in the literature about polyphenol-induced fetal ductal constriction, some case reports deal to specific polyphenol-rich foods, such as chocolate, object of the present study. An example is the publication of Anko and coworkers, where they show a case of ductal constriction in a 34-week pregnant woman who had taken 3 pieces of chocolate (127 mg of polyphenol per piece) after 34 weeks^39^.

In conclusion, this study shows further knowledge on the acute effect of maternal ingestion of cocoa on fetal ductus arteriosus, demonstrating that even a single dose of concentrated cocoa may cause ductus arteriosus constriction similar to its positive control—indomethacin, and corroborating the conceptual hypothesis. Additional clinical studies are necessary to establish dose ranges and safety in the human setting.

## Data Availability

The datasets generated during and/or analyzed during the current study are available from the corresponding author on reasonable request.
